# The response of a boreal deep-sea sponge holobiont to acute thermal stress

**DOI:** 10.1038/s41598-017-01091-x

**Published:** 2017-05-22

**Authors:** R. Strand, S. Whalan, N. S. Webster, T. Kutti, J. K. H. Fang, H. M. Luter, R. J. Bannister

**Affiliations:** 1 Institute of Marine Research, Bergen, Norway; 2 Department of Biology, University of Bergen, Bergen, Norway; 3 Marine Ecology Research Centre, Southern Cross University, Lismore, NSW 2478 Australia; 4 Australian Institute of Marine Science, Townsville, Australia; 5 Australian Centre for Ecogenomics, University of Queensland, Queensland, Australia; 6 Victoria University of Wellington, Wellington, New Zealand

## Abstract

Effects of elevated seawater temperatures on deep-water benthos has been poorly studied, despite reports of increased seawater temperature (up to 4 °C over 24 hrs) coinciding with mass mortality events of the sponge *Geodia barretti* at Tisler Reef, Norway. While the mechanisms driving these mortality events are unclear, manipulative laboratory experiments were conducted to quantify the effects of elevated temperature (up to 5 °C, above ambient levels) on the ecophysiology (respiration rate, nutrient uptake, cellular integrity and sponge microbiome) of *G. barretti*. No visible signs of stress (tissue necrosis or discolouration) were evident across experimental treatments; however, significant interactive effects of time and treatment on respiration, nutrient production and cellular stress were detected. Respiration rates and nitrogen effluxes doubled in responses to elevated temperatures (11 °C & 12 °C) compared to control temperatures (7 °C). Cellular stress, as measured through lysosomal destabilisation, was 2–5 times higher at elevated temperatures than for control temperatures. However, the microbiome of *G. barretti* remained stable throughout the experiment, irrespective of temperature treatment. Mortality was not evident and respiration rates returned to pre-experimental levels during recovery. These results suggest other environmental processes, either alone or in combination with elevated temperature, contributed to the mortality of *G. barretti* at Tisler reef.

## Introduction

A changing global climate is predicted to significantly impact marine environments^1–3^ and well-studied shallow tropical coral reefs provide evidence of the sensitivity of marine ecosystems to increases in sea surface temperature (SST)^4^. These climate effects are particularly evident for sessile invertebrate species, which often exhibit narrow ranges of thermal tolerance^5–7^. Shallow polar seas, although less well studied, are also susceptible to elevated SST^8–10^. However, a large knowledge gap exists for how increasing SST will affect deep-sea boreal ecosystems^11^ and it is currently unknown whether depth can buffer the impacts of rising SST^12^.

Many deep-sea ecosystems are dominated by sponges which can contribute to >90% of the invertebrate biomass^13, 14^. Deep-sea sponge beds create complex habitats that support high species diversity, including commercially important fish species^15^. In addition to their conspicuous biomass and role in habitat formation, deep-sea sponges influence ecosystem dynamics via their role in nutrient cycling^16–20^. For instance, in oceanic regions with high sponge biomass, sponge-associated microorganisms are thought to play a major role in oceanic nitrogen cycling^21^.

Recent mass mortalities events of sponges in shallow-water Mediterranean ecosystems coincided with sudden increases in seawater temperature (1–4 °C above mean summer temperatures)^22–24^. Similar mass mortalities of the ecologically important deep-water sponge *Geodia barretti* were also observed at Tisler Reef, a cold water coral reef along the Norwegian shelf^25^. Concomitant with *G. barretti* mortality (i.e. in 2006 and 2008), thermal increases of up to 4 °C over a 24 h period (increasing from approximately 8 °C to 12 °C) at depths of 70–160 m were recorded on Tisler Reef in southern Norway^25^. Seawater temperatures remained highly variable for up to 7 weeks, but only exceeded 12 °C continuously for no more than 12 days during this period before cooling. Long term temperature data indicated that these thermal anomalies in 2006 and 2008 were uncharacteristic^25^. However, to date, the cause of *G. barretti* mortalities has not been empirically determined and no experimental research has ascertained the thermal sensitivity of this sponge species.

Experimental and field research on shallow water sponge species has documented species-specific responses to elevated SST. Some photosynthetic sponge species show evidence of photosymbiont loss in response to increased sea surface temperatures (>30 °C) which results in sponge “bleaching”^26–29^. The microbiome of non-photosynthetic species can also be disrupted by elevated SST (5 °C above mean summer temperatures) with significant adverse consequences for the host sponge^30, 31^. Increased SST between 2 and 5 °C above ambient summer mean temperatures can also impact feeding behaviour, energetic profiles, gene expression profiles, chemical defences and cause host-symbiont molecular interactions to breakdown^31–35^. A recent analysis of shallow water Great Barrier Reef sponges demonstrated that increase SST of 4 °C above ambient summer mean temperatures decreased the survival of all studied species and resulted in increased levels of tissue necrosis and bleaching, elevating respiration rates and decreasing photosynthetic rates^29^. Importantly however, elevated partial pressure of carbon dioxide *p*CO_2_ was found to exacerbate temperature stress in heterotrophic species, but mitigate temperature stress in phototrophic species. Numerous other studies have explored the combined effects of elevated temperature and *p*CO_2_^36–39^, although most have focussed on sponge bioerosion, which is generally reported to increase with elevated SST and *p*CO_2_. Conversely, other studies have reported sponge tolerance to future predicted climate change conditions with survival, growth, and secondary metabolite biosynthesis of some species unaffected by SST elevated 3 °C above present-day summer-maxima^40, 41^.

At a population level, increased seawater temperatures can also influence sponge life histories linked to reproduction and offspring (larvae), including disruptions to reproductive phenology, planktonic larval competencies, environmental larval settlement cues, and the incursion of sub-lethal molecular costs on larvae^7, 42–45^. Links between elevated seawater temperature and disease also represents a further population level impact to sponges^46^.

*Geodia barretti* is a common deep water sponge that forms dense aggregations in benthic habitats associated with Norwegian fjords and the continental shelf^13, 47^. We aimed to replicate the thermal event that occurred on Tisler Reef to quantify the effect of thermal stress on *G. barretti* and whether this stressor alone is responsible for the reported mass mortality of *G. barretti* individuals on Tisler reef. One year old *G. barretti* explants were exposed to acute thermal stress for 14 days (simulating the exposure time period for the recorded highest temperature period) followed by a 2-month recovery period. Responses associated with physiology (respiration rate, nutrient utilisation and energetics), cellular stress (lysosomal membrane stability) and microbial symbiosis (community shifts) were measured.

## Materials and Methods

### Study species and collection of specimens

Individually cultivated explants (n = 120) of *G. barretti* were collected using the RV Håkon Mosby, from a sponge cultivation site in Oggdalsfjorden^48^. Explants, approximately 4 cm^3^, were prepared from whole sponges and left to heal and grow for 12 months in commercial mussel lanterns at a depth of 170 m. During collection, explants were placed into five 50 L cooler boxes (25 explants per cooler) filled with seawater from 170 m and transported for 1 hr to the deep-sea ecology laboratory at the Institute of Marine Research in Austevoll, Norway. They were maintained in 500 l flow-through tanks supplied with unfiltered seawater from Langenuen Fjord, which was pumped from a depth of 160 m. Sponge explants were acclimated to laboratory conditions in 500 l tanks for 2 weeks prior to being placed into 50 l experimental tanks for a further 5 d acclimation period. These experimental tanks were supplied with sand filter seawater from a depth of 160 m. During the experimental period, no additional feeding was offered since this sand-filtered sea water contains abundant particles <10 μm that can serve as food for sponges^49^.

### Experimental design

Experimental conditions aimed to simulate the acute thermal stress event (7–12 °C) which occurred at Tisler Reef, Norway, in 2006 and 2008^25^. To quantify the acute effect of elevated seawater temperature on the physiology, nutrient utilisation, tissue energetics, cellular integrity and microbial symbiosis of *G. barretti*, explants were exposed to a rapid elevation in seawater temperature, equivalent to 2 °C, 4 °C and 5 °C above ambient at 170 m where the donor sponges were sampled. The corresponding temperature treatments were therefore maintained at 7 °C (7.15 ± 0,28 °C S.D; median of 7.18 °C), 9 °C (8.77 ± 0,31 °C S.D; median of 8.88 °C), 11 °C (11.25 ± 0,29 °C S.D; median of 11.30 °C) and 12 °C (12.31 ± 0,22 °C S.D; median of 12.30 °C). Each temperature treatment had replicate tanks (n = 5) with 6 explants per tank to allow for multiple sampling over time. Temperatures were maintained in the experimental tanks using separate heat exchangers (Aqua Logic Model # CH5536VHD1). Temperatures were increased at 0.4 °C per h, with the 12 °C treatment being reached after 12 h and all tanks maintained at treatment temperatures for 14 d. After exposure to elevated temperature for 14 d, the remaining sponges had a recover period of 65 d at 7 °C (i.e. defined as the recovery period). Therefore, sponges were sampled after 12 h, 24 h, 7 d, 14 d and 79 d.

Respiration and nutrient utilisation were measured repeatedly on the same five sponge explants for each experimental treatment and at each of the sampling time points. In addition, one sponge was sacrificed per tank, at each time point, to assess tissue energetics, the stability of lysosomal membranes and the sponge-associated microbiome.

### Respiration rates

Respiration was measured repeatedly throughout the experimental period following well established methodology^48^. Briefly, each explant was held in an individual acrylic 500 ml cylindrical respiration chamber. To ensure non-static conditions each respiration chamber was fitted with a mechanised stirring bar operating at a rate of 500 rpm. Each chamber was placed into an experimental tank to maintain the experimental temperature during incubations. Respiration was measured as the change in oxygen concentration in the respiration chamber using a calibrated oxygen optode (PreSens) during a 1 h incubation. Oxygen concentrations were also measured in empty respiration chambers (n = 3 for each treatment) to control for oxygen consumption by seawater microorganisms, which was close to zero, and these differences were corrected in the final calculations. After measurement of respiration, the volume and wet weight of each explant was calculated. Explant volume was determined from measurements of the longest axis of length, width and height and wet weight was determined by placing the sponge in a beaker with water on a scale. Wet weight was converted to dry weight using a conversion ratio of 0.2. This conversion ratio was calculated by weighing the wet weight of 27 whole *G. barretti* sponges that were then subsequently dried in an oven at 60 °C to constant weight. The wet weight and dry weight ratio of these sponges were then calculated (Supplementary Fig. 1). Respiration rate of explants was calculated as: (oxygen concentration at start − oxygen concentration at final time of incubation) * (volume of respiration chamber)/(sponge dry weight/time of incubation).

### Nutrient utilisation

Water samples (20 ml) were collected using sterile syringes before and after closed-respiration incubations to measure net nitrite + nitrate (NO_2_^−^ + NO_3_^−^), phosphate (PO_4_^3−^) and silicate (SiO_4_^4−^) fluxes. Nutrient samples were preserved with chloroform and stored at 4 °C. Samples for NO_2_^−^ + NO_3_^−^, PO_4_^3−^ and SiO_4_^4−^ were determined using standard methods adapted for an auto-analyser^50^.

### Tissue sampling for analysis of energetics, lysosomal membrane stability and microbial symbiosis

Tissue samples were collected using a sterile scalpel from explants at each experimental time point. Tissue sub-samples (1 cm^3^) were excised from the sponge choanosome to reduce the risk of surface contaminants associated with the outer cortex. Samples for energetics and microbiology were snap-frozen in liquid nitrogen prior to storage at −80 °C. The lysosomal membrane stability assay was performed on freshly collected tissue immediately after sponge dissection.

### Tissue energetics–elemental composition of Carbon, Hydrogen, Oxygen, Nitrogen and Sulphur

To quantify changes in tissue energetics associated with thermal stress, elemental analysis was undertaken by a commercial lab–OEA Labs, UK. Frozen tissue samples were freeze dried, manually homogenised using a mortar and pestle, and the proportions of carbon, hydrogen, oxygen, nitrogen and sulphur (CHONS) were analysed following established methodology^48^. Briefly, tissue samples were flash combusted at 1800 °C in helium with a controlled dosage of oxygen. A chromatograph with a thermal conductivity detector was used to separate and quantify the resulting combustion gases. For each sample, percentages (by mass) of carbon, hydrogen, nitrogen and sulphur were combined with the percentage of ash and oxygen as determined by calculating the remaining organic fraction. The total energy content in the sponge tissues (expressed as the higher heating value, HHV), were calculated from the elemental measurements using the standard formula^51^:

$$\begin{array}{rcl}{\rm{HHV}}({\rm{J}}/{\rm{mg}}) & = & (0.3491\times {\rm{C}})+(1.1783\times {\rm{H}})-(0.1034\times {\rm{O}})\\  &  & -(0.0151\times {\rm{N}})+(0.1005\times {\rm{S}}).\end{array}$$

### Lysosomal membrane stability

Lysosomal membrane stability was measured on freshly cut sponge tissue following standard methods^52, 53^ modified for sponges^54^. Approximately 1 cm^3^ of choanosome was excised from each explant, rinsed and stored in seawater for a maximum of 1 h. Tissue was further minced into 1 mm^3^ pieces which were placed into a single well of a 24-well culture plate with 1 ml of Ca^2+^/Mg^2+^ free saline (CMFS) and agitated in a shaker at 120 rpm for 30 min. The tissue homogenate was filtered through a 40 µm nylon mesh into a 2 ml microcentrifuge tube. Samples were centrifuged at 300 g, 10 °C for 5 min, before the supernatant was discarded and the pellets resuspended in 1 ml CMFS and centrifuged again at 300 g for 5 min. The supernatant was again discarded and the pellet resuspended in 50 µl CMFS. Fifty µl of neutral red solution (N4638, Sigma-Aldrich) was added and mixed in the sample, following storage for 1 h in a light protected humidified chamber at room temperature. A small aliquot of the cell suspension was placed onto a microscope slide and viewed under a light microscope (400x magnification). A total of 50 cells were assessed for lysosomal integrity. Cells with neutral red contained discretely within the lysosomes were scored as stable, while cells with neutral red leaking into the cytoplasm were scored as destabilised.

### DNA Extraction, Sequencing and Processing

To assess community shifts in the microbiome of *G. barretti*, across temperature treatments, genomic DNA was extracted using the PowerSoil DNA Isolation Kit (MoBio Laboratories, Inc.), following the manufacturer’s protocol. Samples were sequenced as part of the Earth Microbiome Project (EMP)^55^ on the Illumina platform using the bacterial primers 515F/860R and standard protocols^56^.

Fastq sequences provided by the EMP (quality-filtered and de-multiplexed) were processed using Mothur v.1.35.1^57^. Sequences were filtered using the following parameters: average quality score = 30, window size = 5 bases, maximum ambiguity = 0 and maximum number of homopolymers = 8, and trimmed to 100 bp. Unique sequences were aligned against a trimmed (V4 region) SILVA database and chimeric sequences identified by UCHIME (Edgar *et al*.^58^) were removed. Remaining sequences were then grouped into operational taxonomic units (OTU) at 97% sequence similarity using the furthest neighbour clustering method. Representative sequences were classified based on the SILVA database, using a minimum cut-off of 60%. Singletons and doubletons (OTUs formed by one or two sequence(s), respectively) were removed and samples were rarefied to 11,440 reads prior to statistical analyses. Processed sequences from the EMP and meta-data are available via the following portal (http://qiita.microbio.me/) under study number 10533.

### Statistical Analysis

Statistical analyses were carried out using the SYSTAT statistical package within SIGMAPLOT v11.2 and SPSS Statistics v24. Data analysed using Repeated-measures ANOVA and ANOVA models were checked for homogeneity of variances and normality using standardised residuals versus predicted value plots and Q–Q plots of residuals. All data conformed to the assumptions of ANOVA. All data are presented as mean ± 1SE unless stated otherwise. Statistical analysis for respiration and nutrient fluxes (nitrite/nitrate production, silicate retention and phosphate production) were analysed using a two-factor (time × treatment) repeated measures analysis of variance (ANOVA). Statistical significance for repeated measures ANOVA models were estimated from the Greenhouse-Geisser adjusted probability of the within- subject (time) variance–covariance matrix^59^. Tissue energetic levels and lysosomal membrane stability were analysed using a two-factor (time × treatment) Analysis of Variance (ANOVA). Visual comparisons of the *G. barretti* microbiome were undertaken using principal coordinate analysis (PCO), while PERMANOVA was used to statistically test differences in community structure between temperature treatments. All microbial statistical analyses were based on Bray-Curtis distances of square root transformed data and performed using PRIMER 6/PERMANOVA + v1.0.2 (Plymouth, UK).

### Results

No sponges, from any treatment, showed visible signs of stress (i.e. signs of tissue loss, necrosis or change in colour) throughout the experimental and recovery periods.

### Respiration rate

**Figure 1:**
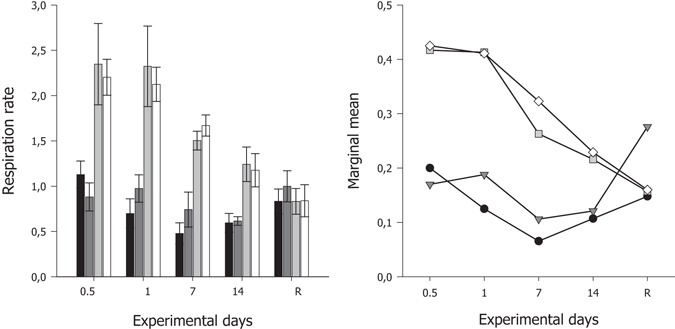
*Left panel*–Mean respiration rate (μmol O_2_ g (tissue DW)^−1^ h^−1^) ± S.E. (n = 5) of cultivated *G. barretti* explants exposed to four different temperature levels (7 °C–Black bar, 9 °C–Dark grey bar, 11 °C–Light grey bar & 12 °C–White bar) over a 14 d experimental period. R on the x-axis is respiration at 65 d post exposure (the recovery period), with explants being maintained at ambient temperatures (7 °C) after the 14 d experimental period, irrespective of prior temperature exposure. *Right panel*–Interaction plot of estimated marginal means of respiration rates calculated for each temperature (7 °C–Black circles, 9 °C–Dark grey triangles, 11 °C–Light grey squares & 12 °C–White diamonds) at each exposure day and recovery time period (R).

A significant interaction between the main effects of temperature and duration of exposure were responsible for the differences in respiration rates of *G. barretti* under the experimental treatments (*P* < 0.001, Table 1, Fig. 1). Of interest in the interaction are the trends at exposure temperatures of 11 °C and 12 °C, where marginal means (the mean of the marginal/probability distribution) for *G. barretti* individuals were consistently higher, at each sampling time point, than the marginal means for *G. barretti* individuals exposed to 7 °C and 9 °C (with the exception of the recovery period, Fig. 1). Respiration rates for *G. barretti* individuals exposed to 11 °C (2.35 ± 0.45 μmol O_2_ g(tissue DW)^−1^ h^−1^) and 12 °C (2.20 ± 0.20 μmol O_2_ g(tissue DW)^−1^ h^−1^) were more than 2 times higher than *G. barretti* individuals exposed to 7 °C (1.13 ± 0.15 μmol O_2_ g(tissue DW)^−1^ h^−1^) and 9 °C (0.88 ± 0.16 μmol O_2_ g(tissue DW)^−1^ h^−1^) (Fig. 1). The effect of temperature on respiration was different at each time point, particularly at the higher temperature treatments (11 and 12 °C). Interestingly, respiration rates returned to ambient levels after 65 d recovery, ranging between 0.83–1.00 μmol O_2_ g(tissue DW)^−1^ h^−1^ (Fig. 1).

**Table 1:** Summary statistics of repeated measures two-factor ANOVA models for respiration rates and nutrient dynamics of *G. barretti* explants exposed to different experimental temperature treatments (7, 9, 11 and 12 °C) over a 14 d period, followed by a two month recovery period (12 h, 24 h, 7 d, 14 d and Recovery).

Source	df	MS	*F*	P
**Respiration**				
*Within subjects*				
Day	1.828	4.891	17.388	<0.001
Day × Temperature	5.483	1.311	4.660	0.003
Error	25.586	0.281		
*Between subjects*				
Temperature	3	4.940	8.796	0.002
Error	14	0.562		
**Nitrogen efflux**				
*Within subjects*				
Day	2.036	0.002	4.396	0.002
Day × Temperature	6.107	0.001	1.802	0.131
Error	30.535	0,000		
*Between subjects*				
Temperature	3	0.002	4.970	0.014
Error	15	0.000		
**Phosphate efflux**				
*Within subjects*				
Day	2.814	0.00003	17.678	<0.001
Day × Temperature	8.452	0.00001	10.027	<0.001
Error	42.258	0.000001		
*Between subjects*				
Temperature	3	0.00002	12.643	<0.001
Error	16	0.000001		
**Silicate flux**				
*Within subjects*		Ms	F	P
Day	2.636	0.00003	4.017	0.017
Day × Temperature	7.908	0.00001	1.509	0.186
Error	39.541	0.00001		
*Between subjects*				
Temperature	3	0.00001	0.555	0.653
Error	15	0.00002		

Significance values are based on the Greenhouse-Geisser corrections.

### Nutrient utilisation

#### NO_2_^−^ + NO_3_^−^

**Figure 2:**
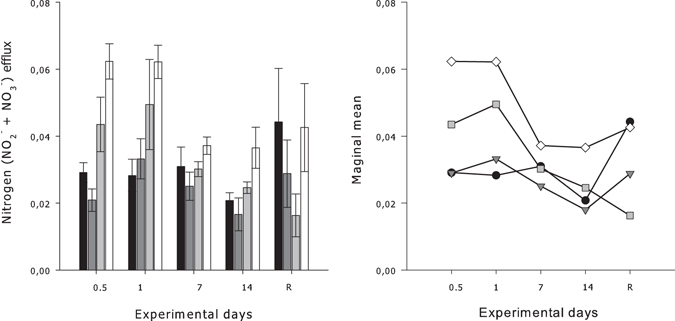
*Left panel*–Mean net nitrogen efflux rate (μmol NO_2_^−^ + NO_3_^−^ g(tissue DW)^−1^ h^−1^) ± S.E. (n = 5) by *G. barretti* explants exposed to four different temperature levels (7 °C–Black bar, 9 °C–Dark grey bar, 11 °C–Light grey bar & 12 °C–White bar) over a 14 d experimental period. R on the x-axis is respiration at 65 d post exposure (the recovery period), with explants being maintained at ambient temperatures (7 °C) after the 14 d experimental period, irrespective of prior temperature exposure. *Right panel*–Interaction plot of estimated marginal means of nitrogen (NO_2_^−^ + NO_3_^−^) flux rates calculated for each temperature (7 °C–Black circles, 9 °C–Dark grey triangles, 11 °C–Light grey squares & 12 °C–White diamonds) at each exposure day and recovery time period (R).

The production of NO_2_^−^ + NO_3_^−^ by *G. barretti* explants was significantly affected by both temperature (*P* = 0.014, Table 1) and the duration of exposure (*P* = 0.002, Table 1) over the experimental period. Trends of increased marginal means for *G. barretti* explants exposed to water temperatures 4 and 5 °C above ambient temperatures (11 and 12 °C, respectively) were consistently higher than G. *barretti* explants exposed to ambient temperature (7 °C) for the first 24 h (Fig. 2). The highest production of NO_2_^−^ + NO_3_^−^ in *G. barretti* explants occurred during the first 24 h for explants exposed to 12 °C (0.062 ± 0.005 μmol g(tissue DW)^−1^ h^−1^) and was twice as high as NO_2_^−^ + NO_3_^−^ production from explants at ambient temperatures (0.029 ± 0.003 μmol g(tissue DW)^−1^ h^−1^). These elevated responses reduced during the remainder of the experiment. The production of NO_2_^−^ + NO_3_^−^ by *G. barretti* explants varied between 0.016–0.044 μmol g(tissue DW)^−1^ h^−1^ during the 65 d recovery period.

#### Phosphate

**Figure 3:**
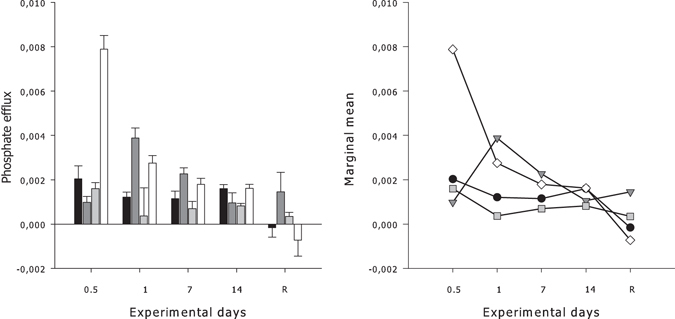
*Left panel*–Mean net phosphate efflux rate (μmol PO_4_^3−^ g(tissue DW)^−1^ h^−1^) ± 1 S.E. (n = 5) by *G. barretti* explants exposed to four different temperature levels (7 °C–Black bar, 9 °C–Dark grey bar, 11 °C–Light grey bar & 12 °C–White bar) over a 14 d experimental period. R on the x-axis is respiration at 65 d post exposure (the recovery period), with explants being maintained at ambient temperatures (7 °C) after the 14 d experimental period, irrespective of prior temperature exposure. *Right panel*–Interaction plot of estimated marginal means of PO_4_^3−^ flux rates calculated for each temperature (7 °C–Black circles, 9 °C–Dark grey triangles, 11 °C–Light grey squares & 12 °C–White diamonds) at each exposure day and recovery time period (R).

A significant interaction between the temperature and duration of exposure (*P* < 0.001, Table 1) contributed to the variability in PO_4_^3−^ production by *G. barretti* explants (Fig. 3). Marginal means were highest for *G. barretti* explants during the first 12 h of exposure to 12 °C, however, after 14 d of exposure the marginal means were similar between experimental treatments (Fig. 3). The highest production of PO_4_^3−^ occurred within the first 12 h of exposure at the highest temperature treatment (0.008 ± 0.001 μmol g(tissueDW)^−1^ h^−1^) and was highly variable between treatments at the recovery time point ranging between −0.001–0.001 μmol g(tissue DW)^−1^ h^−1^ (Fig. 3).

#### Silicate

**Figure 4:**
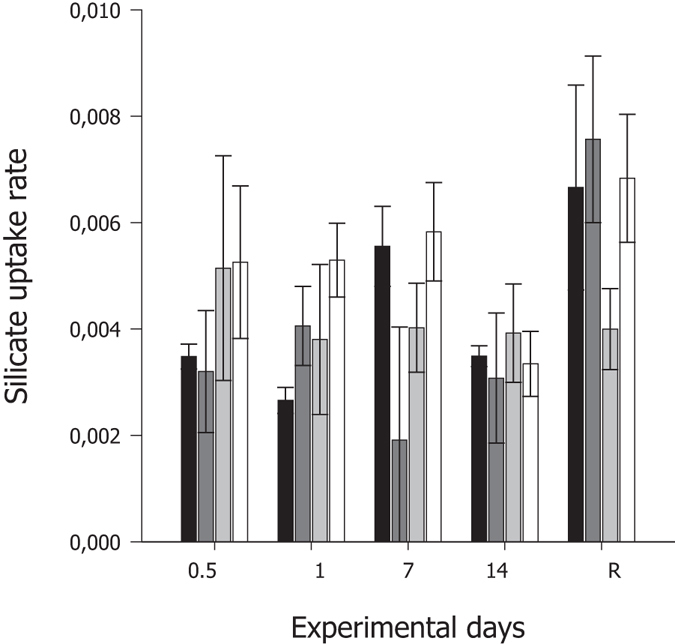
Mean net silicate uptake rate (μmol SiO_4_^4−^ g(tissue DW)^−1^ h^−1^) ± S.E. (n = 5) by *G. barretti* explants exposed to four different temperature levels (7 °C–Black bar, 9 °C–Dark grey bar, 11 °C–Light grey bar & 12 °C–White bar) over a 14 d experimental period. R on the x-axis is respiration at 65 d post exposure (the recovery period), with explants being maintained at ambient temperatures (7 °C) after the 14 d experimental period, irrespective of prior temperature exposure.

No significant interaction between temperature exposure and duration of exposure (*P* = 0.123, Table 1) was evident for the production/consumption of Si by *G. barretti* explants (Fig. 4). Furthermore, the production/consumption of SiO_4_^4−^ was not significantly influenced by the main effects of elevated temperature over the production period (*P* = 0.651, Table 1). However, duration of exposure had a significant effect on the production/consumption of SiO_4_^4−^ by *G. barretti* explants (P = 0.003, Table 1), ranging between 0.002–0.005 μmol g(tissue DW)^−1^ h^−1^ during the first 24 h to as high as 0.008 ± 0.001 μmol g (tissue DW)^−1^ h^−1^ during the recovery period (Fig. 4).

### Lysosomal membrane stability

**Figure 5:**
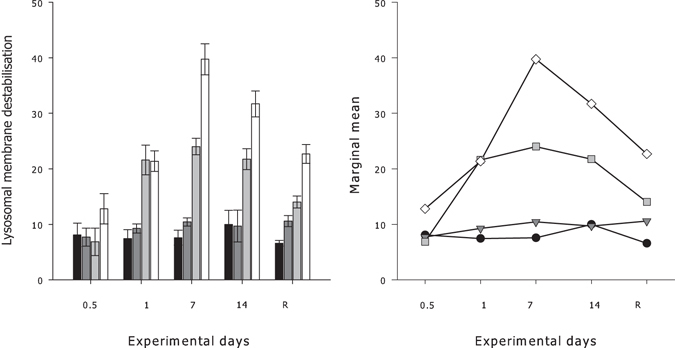
*Left panel*–Mean percentages (%) of cells with destabilised lysosomal membranes ± S.E. (n = 5) of *G. barretti* explants exposed to four different temperature levels (7 °C–Black bar, 9 °C–Dark grey bar, 11 °C–Light grey bar & 12 °C–White bar) over a 14 d experimental period. R on the x-axis is respiration at 65 d post exposure (the recovery period), with explants being maintained at ambient temperatures (7 °C) after the 14 d experimental period, irrespective of prior temperature exposure. *Right panel*–Interaction plot of estimated marginal means of lysosomal membrane destabilisation calculated for each temperature (7 °C–Black circles, 9 °C–Dark grey triangles, 11 °C–Light grey squares & 12 °C–White diamonds) at each exposure day and recovery time period (R).

Exposure temperature and duration of exposure both showed significant main effects on the destabilisation of lysosomal membranes for *G. barretti* explants (Table 2). A significant interaction between duration of exposure and exposure temperature drove the destabilisation of lysosomal membranes of *G. barretti* (*P* < 0.001, Table 2, Fig. 5). Of interest in the interaction are the trends at exposure temperatures of 11 °C and 12 °C, where marginal means for *G. barretti* individuals were consistently higher at each sampling time point after 12 h than the marginal means for *G. barretti* individuals exposed to 7 °C and 9 °C (Fig. 5). The proportion of destabilised lysosomal membranes for *G. barretti* individuals exposed to 11 °C (24 ± 1.48%) and 12 °C (39.7 ± 2.8%) were 2–5 times higher than *G. barretti* individuals exposed to 7 °C (7.6 ± 1.3%) and 9 °C (10.4 ± 0.7%) (Fig. 5). The effect of temperature on destabilisation of lysosomal membranes was different at each measurement time point, particularly at the higher temperature treatments (11 and 12 °C). Interestingly, 65 d of recovery resulted in a greater number of destabilised lysosomal membranes for explants exposed to 11 °C and 12 °C than for the 7 °C and 9 °C (Fig. 5).

**Table 2:** Summary statistics of two-factor ANOVA for lysosomal destabilisation and tissue energetics of *G. barretti* explants exposed to different experimental temperature treatments (7, 9, 11 and 12 °C) over a 14 d period, followed by a two month recovery period (12 h, 24 h, 7 d, 14 d and Recovery).

Source	df	MS	*F*	P
**Lysosomal destabilisation**				
Day	4	400.703	21.139	<0.001
Temperature	3	1668.461	88.019	<0.001
Day × Temperature	12	133.526	7.044	<0.001
Residual	80	18.956		
**Tissue energetics**				
Day	1	1.704	1.609	0.214
Temperature	3	0.910	0.859	0.472
Day × Temperature	3	0.111	0.105	0.957
Residual	32	1.059

### Energetics

Energetic content of sponges expressed as the higher heating value exposed to the four temperature treatments for 14 d (ranging between 18.6–19.8 J mg tissue^−1^) was similar to *G. barretti* explants sampled after 65 d recovery across the four temperature treatments (ranging between 18.8–19.1 J mg tissue^−1^), with no significant changes across treatments or time of sampling (Table 2).

### Microbial community analyses

**Figure 6:**
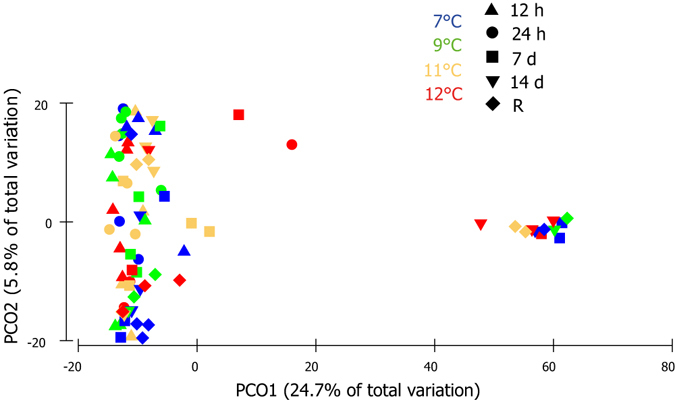
Principal coordinate analysis (PCO) of the bacterial OTUs from *G. barretti* explants exposed to four different temperature levels (see attached legend) over a 14 d experimental period. R on the legend represents 65 d post exposure (the recovery period), with explants being maintained at ambient temperatures (7 °C) after the 14 d experimental period, irrespective of prior temperature exposure.

Of the 100 *G. barretti* samples sequenced, 17 produced insufficient reads and were removed from the analysis (Supplementary Table 1). A total of 7,991 OTUs were identified (97% sequence similarity) across the remaining 83 *G. barretti* samples, spanning 20 different bacterial phyla and one archaeal phyla (Supplementary Fig. 2). PCO analysis explained 30.5% of the total variation in the first two factors (Fig. 6). The majority of samples clustered together, with no significant difference in the microbiome between temperature treatments (PERMANOVA, Pseudo-F_3,82_ = 1.07, p = 0.290). However, 12 samples representing all four temperature treatments grouped together separately to the right of the ordination (Fig. 6). Spearman-Rank correlation (ρ ≥ 0.6) revealed OTU28 (*Thaumarchaeota*) and OTU40 (unclassified bacteria) were responsible for the separation, with these 12 samples all having higher abundances of both OTUs. Of the 12 samples clustering separately, none were from the 12 h or 24 h time point, which likely contributed to the significant difference detected over time (PERMANOVA, Pseudo-F_4,82_ = 1.92, p = 0.008).

Taxonomic assignment of the OTUs revealed that the microbiome of *G. barretti* is dominated by unclassified bacteria which comprised more than 50% of the total community (relative abundance 51.6% ± 6.4%), regardless of treatment or time point (Supplementary Fig. 2). *Archaea* (13.9% ± 5.6%) and *Gammaproteobacteria* (13% ± 3.2%) were also highly abundant. Unclassified *Proteobacteria* (5.6% ± 1.1%), *Acidobacteria* (5.4% ± 1.5%) and *Alphaproteobacteria* (2.2% ± 0.8%) were moderately abundant and numerous other phyla were represented at low abundance (Supplementary Fig. 2).

## Discussion

*Geodia barretti* is an abundant, habitat forming sponge in deep-water North Atlantic habitats^13, 47, 60^. The mass mortality of *G. barretti* at Tilser Reef^25^ coincided with down-welling events (2006 and 2008) resulting in a rapid rise in bottom water temperature. It was therefore hypothesised that thermal stress contributed to the mass mortality event. However, experimental research exposing *G. barretti* to temperatures, up to 5 °C above ambient temperature for 14 days, only induced sub-lethal physiological and cellular stress.

External morphological tissue degradation (i.e. tissue necrosis, cell loss, pinacoderm or choanoderm colour changes) is a common sign of compromised sponge health^30, 31^ and was observed in *G. barretti* at Tisler Reef during the recent mass mortality event^25^. However, despite exposure of sponge explants to temperatures 2–5 °C above ambient mean seawater temperature, in the present study, no visible signs of stress were evident in any of the samples although some sub-lethal effects on physiology and cellular integrity were noted.

Both elevated temperature and length of exposure period contributed to variability in *G. barretti* respiration rates and net nutrient fluxes, although respiration rates were within the range reported from other studies^47, 48, 61^. Environmental temperature can be an important driver of organism respiration with increased respiration being experimentally demonstrated in a range of thermally stressed marine invertebrates^62–64^. While time and temperature had a significant effect on *G. barretti* respiration during the 14-day temperature exposure, respiration returned to ambient levels during the recovery phase, highlighting a capacity for the sponge to manage its metabolism when exposed to acute pulses of temperature stress. Reduction in energetic content (i.e. CHONS analysis) did not occur in sponges at the higher temperature treatments, therefore, increased respiration (i.e. indications of increased energy demand) may occur at the expense of other physiological functions, or life history traits such as reproduction (not assessed here). Higher metabolic rates and increased investment in somatic maintenance occur as trade-offs to growth and reproduction for some marine invertebrates exposed to sub-optimal environments^65^. In particular, temperature increases can compromise growth and reproduction in marine invertebrates^3, 66–68^. However, given the short exposure periods over which elevated respiration occurred in *G. barretti*, and the capacity for rapid recovery, it would seem unlikely that added energy investment in respiration would be at the expense of reproduction or growth. Further work would be valuable to establish if physiological trade-offs among organism maintenance, reproduction and growth would occur following longer periods of temperatures stress for *G. barretti*. Increased temperature over time also lead to changes in net nitrogen flux rates from *G. barretti* during the experimental period. These increased flux rates correlated with the increased oxygen flux rates within the same temperature treatments over the experimental period. Therefore, oxygen consumption by the microorganisms undertaking nitrification and other key microbial processes within *G.barretti* ^17^ may also be responsible for a proportion of the elevated oxygen flux that was observed.

Increased temperatures over time also contributed to the destabilisation of lysosomal membranes. The compromised integrity of the lysosomal membranes in explants exposed to 11 and 12 °C, without any sign of mortality, demonstrates that a 4–5 °C temperature increase represents sub-lethal thermal stress. Temperature induced cellular stress, as measured by lysosomal membrane stability, has been reported for other invertebrates, such as mussels exposed to increased temperatures (≥7 °C above ambient)^69^. The process of rapid cell turnover and shedding that occurs in some sponge species^70, 71^ is thought to contribute to reducing cellular damage caused by environmental stress. This biological process potentially provides a pathway for more rapid recovery following sub lethal stressors that result in cellular damage. However, in this study, thermally stressed *G. barretti* also maintained higher levels of destabilised lysosomes even after the 65-day recovery. Cell turnover rates can be highly variable amongst sponge species^71^ and it is plausible that this process occurs over longer time-frames in *G. barretti* than has been documented for tropical sponges*;* this species is a deep-water boreal sponge (cold water) and may therefore exhibit a slower process of cell turnover in comparison to warmer water sponges. This has been demonstrated recently for 4 other cold water sponges (*Spongilla lacustris, Sycon coactum*, *Haliclona mollis* and *Aphrocallistes vastus*)^72^, where cell turn-over rates were up to 1% of their body replaced daily, more than one to two order of magnitude slower than for warmer water sponges^71^.

Symbiotic microbes are critical to the health and stability of the sponge host^72^, playing important roles in nitrogen and sulphur cycling within the holobiont^17, 21^. Environmental stress induced dysbiosis in the microbiome has been reported for a large number of sponge species, particularly in response to elevated temperature^30, 32, 74–77^ where microbial shifts often correspond with declines in host health. Small temperature increases 1–3 °C above ambient mean summer temperatures caused microbial community shifts in *Halichondria bowerbanki* and *Rhopaloeides odorabile* with the simultaneous disappearance of symbionts and the acquisition of other rare microbes commonly associated with coral disease^30, 78^. Whilst most studies have not dissected cause-effect pathways for the microbiome disruption under thermal stress, Fan and colleagues employed expression profiling of the sponge host and functional analysis of the symbiont community to reveal disruptions to host-symbiont nutritional interdependence and a microbiome shift from one with predominantly symbiotic functions to one characterised by opportunistic bacterial functions^76^. However, despite the large body of literature documenting temperature induced shifts in the sponge microbiome, the microbial community in *G. barretti* remained stable across all temperature treatments, with the exception of 12 samples from the later timepoints, where the relative abundance of OTU28 (Thaumarchaetoa) and OTU40 (unknown) increased. The mechanisms driving the increased abundance of these OTUs are unknown and more detailed metagenomic analysis would be required to provide insights into the functional basis of specific OTU increases. Furthermore, the lack of large fluctuations in nutrient cycling across the experimental treatments indicates a highly stable microbiome in both composition and function. Sponge microbiome stability under elevated temperature has also been reported for temperate^79^ and tropical sponges^31^. The temperate sponges *Ircinia fasciculata* and *I. oros* maintained stable microbiomes despite being exposed to experimental temperatures 12 C higher than ambient levels for more than 2 weeks. The tropical sponge *Ianthella basta* also maintained a stable microbiome despite being exposed to 32 °C, 5 °C higher than ambient mean summer temperatures. In contrast to *Ianthella basta* that displayed visual responses to the elevated temperature exposure including blackening of the tissues^31^, *G. barretti* explants remained visually unaffected. These results highlight the tolerance of the *G. barretti* holobiont to short term elevated acute temperature exposures.

## Conclusion

*Geodia barretti* exhibits high thermal tolerance, with no visible adverse effects and a highly stable microbiome even at temperatures 5 °C above ambient. However, rapid elevation from 7 °C to 11–12 °C did induce sub-lethal physiological and cellular stress responses with some sustained effects at the cellular level even after 65 days recovery. The apparent thermal tolerance of *G. barretti* contrasts earlier field observations where mass mortalities of *G. barretti* at Tisler Reef coincided with acute thermal stress events^25^. Given the experimental design closely replicated the thermal profiles that naturally occurred at Tisler Reef, it is unlikely that thermal stress was solely responsible for the observed mass mortalities. Other ecological processes such as low oxygen concentrations, elevated nutrients levels, reduced salinity and disease, may operate in concert with elevated temperature from down welling events, should be explored to provide insight into the cause:effect pathways of *G. barretti* mortality.

## Electronic supplementary material

Supplementary Data
