# Supplementary material for: The response of a boreal deep-sea sponge holobiont to acute thermal stress
Source: Sci Rep. 2017 May 22;7:1660. doi: 10.1038/s41598-017-01091-x (PMC5440399; doi:10.1038/s41598-017-01091-x)
Supplement: Supplementary file 1 — Supplementary Data [file 41598_2017_1091_MOESM1_ESM.pdf]

The response of a boreal deep-sea sponge holobiont to acute thermal stress

Strand R[1,2], Whalan S[3,4], Webster N. S.[5], Kutti T[1] Fang J.K.H[1], Luter H.M.[5,6], Bannister RJ,[1*]
[1]Institute of Marine Research, Bergen, Norway
[2]Department of Biology, University of Bergen, Norway
[3]Central Caribbean Marine Institute, PO Box 37 Little Cayman, KY3-2501, Cayman Islands
[4]Marine Ecology Research Centre, Southern Cross University, Lismore, NSW, 2478, Australia
[5]Australian Institute of Marine Science, Townsville, Australia
[6]Victoria University of Wellington, Wellington, New Zealand
[*]Correspondence to Raymond.Bannister@imr.no

Supplementary material

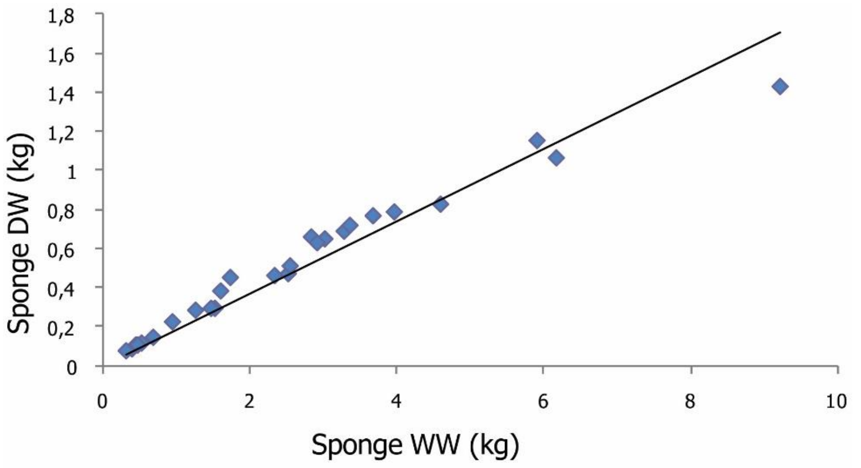

Supplementary Fig. 1: Simple linear regression of wet weight (WW, kg) and dry weight (DW, kg) of *Geodia barretti* (n=27). y = 0,1849x; R^2 = 0,9436.

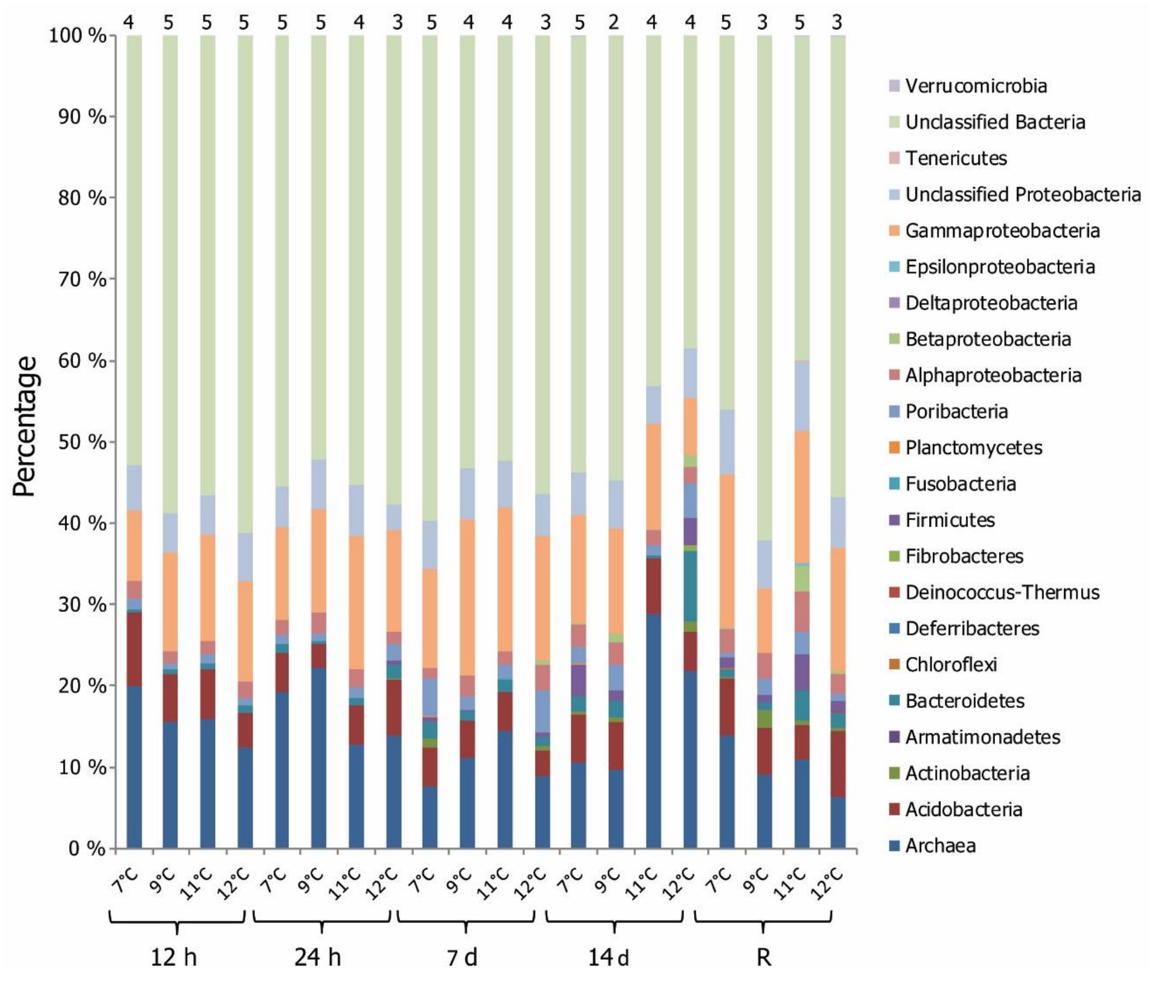

Supplementary Fig. 2: Relative abundance of Archaea and each bacterial phyla, class for Proteobacteria, within each treatment. Replicate individuals per treatment were averaged, with the replicate number denoted at the top of each bar.

Supplementary Table 1. Sample IDs used for sequencing. Samples that had insufficient read counts are denoted in red.

Sample Sequencing ID	Treatment (°C)	Time point
10533.NW1	7	12 h
10533.NW2	7	12 h
10533.NW3	7	12 h
10533.NW4	7	12 h
10533.NW5	7	12 h
10533.NW6	9	12 h
10533.NW7	9	12 h
10533.NW8	9	12 h
10533.NW9	9	12 h
10533.NW10	9	12 h
10533.NW11	11	12 h
10533.NW12	11	12 h
10533.NW13	11	12 h
10533.NW14	11	12 h
10533.NW15	11	12 h
10533.NW16	12	12 h
10533.NW17	12	12 h
10533.NW18	12	12 h
10533.NW19	12	12 h
10533.NW20	12	12 h
10533.NW21	7	24 h
10533.NW22	7	24 h
10533.NW23	7	24 h
10533.NW24	7	24 h
10533.NW25	7	24 h
10533.NW26	9	24 h
10533.NW27	9	24 h
10533.NW28	9	24 h
10533.NW29	9	24 h
10533.NW30	9	24 h
10533.NW31	11	24 h
10533.NW32	11	24 h
10533.NW33	11	24 h
10533.NW34	11	24 h
10533.NW35	11	24 h
10533.NW36	12	24 h
10533.NW37	12	24 h
10533.NW38	12	24 h
10533.NW39	12	24 h
10533.NW40	12	24 h

10533.NW41	7	7 d
10533.NW42	7	7 d
10533.NW43	7	7 d
10533.NW44	7	7 d
10533.NW45	7	7 d
10533.NW46	9	7 d
10533.NW47	9	7 d
10533.NW48	9	7 d
10533.NW49	9	7 d
10533.NW50	9	7 d
10533.NW51	11	7 d
10533.NW52	11	7 d
10533.NW53	11	7 d
10533.NW54	11	7 d
10533.NW55	11	7 d
10533.NW56	12	7 d
10533.NW57	12	7 d
10533.NW58	12	7 d
10533.NW59	12	7 d
10533.NW60	12	7 d
10533.NW61	7	14 d
10533.NW62	7	14 d
10533.NW63	7	14 d
10533.NW64	7	14 d
10533.NW65	7	14 d
10533.NW66	9	14 d
10533.NW67	9	14 d
10533.NW68	9	14 d
10533.NW69	9	14 d
10533.NW70	9	14 d
10533.NW71	11	14 d
10533.NW72	11	14 d
10533.NW73	11	14 d
10533.NW74	11	14 d
10533.NW75	11	14 d
10533.NW76	12	14 d
10533.NW77	12	14 d
10533.NW78	12	14 d
10533.NW79	12	14 d
10533.NW80	12	14 d
10533.NW81	7	R
10533.NW82	7	R
10533.NW83	7	R
10533.NW84	7	R
10533.NW85	7	R
10533.NW86	9	R

10533.NW87	9	R
10533.NW88	9	R
10533.NW89	9	R
10533.NW90	9	R
10533.NW91	11	R
10533.NW92	11	R
10533.NW93	11	R
10533.NW94	11	R
10533.NW95	11	R
10533.NW96	12	R
10533.NW97	12	R
10533.NW98	12	R
10533.NW99	12	R
10533.NW100	12	R
